# Are People Willing to Take Regular COVID-19 Vaccines? Prevalence and Determinants of Hesitancy for Regular COVID-19 Vaccination: A Random Population-Based Survey in Hong Kong

**DOI:** 10.3390/vaccines11081388

**Published:** 2023-08-20

**Authors:** Yan Li, Mengqi Li, Lin Yang, Daniel Bressington, Sau-Fong Leung, Yao-Jie Xie, Jing Qin, Alex Molasiotis, Angela Y. M. Leung

**Affiliations:** 1 School of Nursing, The Hong Kong Polytechnic University, Hong Kong 999077, China; yan-nursing.li@polyu.edu.hk (Y.L.); l.yang@polyu.edu.hk (L.Y.); sau.fong.leung@polyu.edu.hk (S.-F.L.); grace.yj.xie@polyu.edu.hk (Y.-J.X.); harry.qin@polyu.edu.hk (J.Q.); angela.ym.leung@polyu.edu.hk (A.Y.M.L.); 2 Faculty of Health, Charles Darwin University, Darwin 0815, Australia; daniel.bressington@cdu.edu.au; 3 College of Arts, Humanities and Education, University of Derby, Derby DE22 1GB, UK; a.molasiotis@derby.ac.uk

**Keywords:** COVID-19, vaccine, hesitancy, attitude, population-based survey

## Abstract

The emergence of new coronavirus variants and evidence of waning immunity offered by COVID-19 vaccines draw attention to the need for regular vaccination. Vaccine hesitancy is one of the top ten threats to global health. There is a dearth of knowledge on people’s hesitancy to take regular COVID-19 vaccines. This study aimed to investigate the prevalence and determinants of hesitancy for regular COVID-19 vaccination. A population-based, random telephone survey was performed in Hong Kong in April 2022 (*n* = 1213). The age-standardized hesitancy rate for regular COVID-19 vaccines among Hong Kong adults was 39.4% (95% *CI* = 35.3–44.1%), exhibiting a sloping S-shape with age. Regression analyses revealed that females, young adults, self-perceived fair/bad health, low COVID-19 vaccine uptake, and believing there are better ways for prevention of infection were positive determinants of hesitancy for regular vaccination. Vaccine confidence, perceived severity and availability, trust in manufacturers and government, and civic duty inclination were negative determinants. Tailored vaccine promotions are needed for females, young adults, and people perceiving poor health and receiving fewer doses. Information on infection severity, vaccine availability, and trust in suppliers, products, and governments are key attitude-change facilitators to decrease hesitancy for regular COVID-19 vaccination and cope with future pandemics.

## 1. Introduction

The coronavirus disease pandemic of 2019 (COVID-19), caused by the novel severe acute respiratory syndrome coronavirus 2 (SARS-CoV-2), is continuing to result in significant impacts on global health and the economy [1]. A vaccination program serves as a primary preventive measure that has demonstrated effectiveness in controlling and mitigating SARS-CoV-2-related infection and severe illness [2]. The latest evidence based on the global epidemic data showed that new cases, inpatients, and deaths per million people gradually decreased as the rate of COVID-19 vaccine coverage increased, especially when the coverage rate was over 60% [3]. To date, 70.3% of the world population has received at least one dose of a COVID-19 vaccine, and 64.7% has been fully vaccinated based on the statistics from Our World in Data [4]. However, the rate of booster uptake only reaches 34.9% on a global scale [4]. Importantly, despite fully vaccinated rates rising sufficiently to mitigate the COVID-19 pandemic, the emergence of new variants and evidence of waning immunity offered by vaccines necessitates the requirement of regular boosters [5]. However, studies on willingness to take regular COVID-19 vaccines are relatively scattered compared to those for basal dose or single booster administration.

Several circulating variants of concern have led to waves of infections globally including in Hong Kong. The most important of these are (in chronological order): Alpha (B.1.1.7), Beta (B.1.351), Gamma (P.1), Delta (B.1.617.2), and Omicron (B.1.1.529) [6]. In the context of the fifth wave of infections dominated by Omicron that occurred in Hong Kong since late December 2021, booster programs, including the third, fourth, and fifth courses of COVID-19 vaccines, have been consecutively conducted [7]. By 30 September 2022, a vaccine mandate, that the third dose was required for residents aged 12 years or above, was introduced in Hong Kong to increase vaccination levels [8]. The latest data showed that 84.5% of the Hong Kong population aged 12 years or above has received the third dose as a booster [9]. In contrast, the vaccination coverage rate for the fourth or fifth dose offered to Hong Kong residents aged 12 years or above only reaches 25.3% [9]. The rather low voluntary vaccination rates for boosters in Hong Kong raise concerns about people’s hesitancy to take regular vaccines that are needed to address mutations and declining immunity. Understanding the prevalence and determinants of hesitancy for the Hong Kong population to receive regular COVID-19 vaccination is an urgent issue; however, little is known from the extant literature.

Vaccine hesitancy, defined as a delay in acceptance or refusal to vaccinate despite the availability of vaccines, was highlighted by World Health Organization (WHO) as one of the top ten threats to global health [10]. Though more and more people have completed a full course of inoculation, research findings on people’s hesitancy toward regular COVID-19 vaccines are rarely reported. To date, only a handful of studies worldwide have addressed this important issue, and few adopted random sampling strategies in the general population. Notably, most of these studies only evaluated hesitancy toward a single booster rather than regular vaccines, such as the annual influenza vaccine [11,12,13,14,15]. One European study evaluating people’s willingness to take annual COVID-19 vaccines pointed out the impacts of political and religious participation [5]. The other two studies, with samples from the UK and Jordan, identified additional factors determining the hesitancy of regular vaccines, including confidence in vaccines, experiences of vaccine side effects, and availability of vaccination services [16,17]. In addition, emerging studies on the uptake of a booster suggested that COVID-19 vaccine hesitancy was also associated with experiences of SARS-CoV-2 infection, influenza vaccination, attitude toward vaccine efficacy, and trust in authorities, government, healthcare professionals, and pharmaceutical companies [11,12,13,14,15]. Sociodemographics and medical history, i.e., gender, age, educational attainment, employment, health status, and chronic diseases, were also found to be determinants in these studies [11,12,14].

Systematic evidence on vaccine hesitancy in the context of influenza viruses indicated that vaccine hesitancy is complex and induced by multiple factors [18]. The WHO Strategic Advisory Group of Experts (SAGE) on Immunization developed a model to categorize determinants of vaccine hesitancy building on systematically reviewed studies [19]. The WHO SAGE Vaccine Hesitancy Determinants Matrix Model, which incorporates vaccine-specific, individual/group, and contextual influences on attitudes toward vaccines, has been extensively evaluated empirically as a theoretical framework. The model has been previously adopted to assess attitudes toward vaccines in the context of seasonal influenza as well as the COVID-19 pandemic [20]. Besides the determinants for regular or a booster vaccine described above, these studies identified key antecedents associated with COVID-19 vaccines including perceived severity of infection, perceived knowledge sufficiency, personal beliefs, immunity misconception, media/social impacts, and collective responsibility [20,21,22]. The research findings suggest that the WHO SAGE Vaccine Hesitancy Determinants Matrix Model may help to explain and predict people’s decisions to receive regular COVID-19 vaccines, but this has not yet been examined empirically.

Given the continually changing nature of the pandemic and the likelihood of mutations and waning immunity, understanding people’s willingness to take regular COVID-19 vaccines is urgently required to address the very real threat over the long term. Vaccine hesitancy is a potential impediment to future widespread inoculation because it is a long-lasting phenomenon and fluctuates with waves of infection [23]. Notably, the voluntary vaccination coverage rate for boosters in Hong Kong residents is rather low, while little is known about their hesitancy for regular COVID-19 vaccination. Though several studies have assessed the regular COVID-19 vaccine hesitancy in European and Middle Eastern countries, the prevalence and determining factors might be different in Hong Kong given the cultural and contextual differences [5,16,17]. Moreover, some of the prior studies that explored predictors of regular or booster vaccine hesitancy might not comprehensively address potential influencing factors due to the lack of theoretical underpinnings [5,12,17]. Therefore, our study aimed to conduct a random population-based survey in Hong Kong to evaluate the prevalence and determinants of hesitancy for regular COVID-19 vaccination. This study was theoretically guided by the WHO SAGE Vaccine Hesitancy Determinants Matrix Model, as well as a comprehensive literature review of studies that explored predictors of COVID-19 vaccine hesitancy [11,12,13,14,15,16,17,20,21,22]. The findings will contribute to the vaccine policymakers and health program promoters within government health sectors and healthcare professionals, not only for the COVID-19 pandemic but also for potential future pandemics with newly developed vaccines.

## 2. Materials and Methods

### 2.1. Study Design, Sample, and Ethical Considerations

This study was a random population-based telephone survey. Hong Kong residents who were at least 18 years of age and without difficulties in understanding the online survey were eligible to be participants. To minimize sampling bias, telephone numbers were randomly selected from an updated directory that covers all the Hong Kong landlines and mobile numbers. Assuming the proportion of COVID-19 vaccine hesitancy as 50% [24], a minimum sample size of 1068 subjects was required to achieve a precision level of 3% at a confidence level of 95% (Z = 1.96) from the formula: N = Z^2^(P)(1 − P)/Precision^2^ [25].

This study was approved by the Human Subjects Ethics Review Board of the Hong Kong Polytechnic University (HSEARS20210813003). Informed consent was obtained from all participants.

### 2.2. Data Collection

Data was collected by external services provided by a local telephone company under close supervision, during the period of 4 to 23 April 2022. A pool of telephone numbers was first randomly generated using known prefixes assigned to telecommunication service providers under the numbering plan provided by the Office of the Communications Authority. Both landline and mobile numbers were included in the sampling frame with a target ratio of 1:1. For landline samples, if more than one eligible respondent was available in the sampled household during the call, the selection was made using the “next birthday rule”, whereby the person with the soonest birthday among eligible respondents available at that call time was selected for interview [25]. For the mobile samples, respondents were asked whether they were of age 18 or above. Non-responding numbers remained in the pool for additional attempts on separate occasions. If a number did not respond after a total of five attempts, it was removed from the telephone pool and recorded as “no answer”. The total number of call attempts was 40,864. Duplicate and invalid numbers were eliminated according to computer and manual dialing records to produce the final sample.

### 2.3. Measures

The primary outcome of this study was the hesitancy of regular COVID-19 vaccination. It was assessed by the question “If the COVID-19 vaccine needs (or is suggested by health professionals) to be taken regularly every year in the future, will you take it?” with responses of Yes, No, or Not sure. Options for No or Not Sure were categorized to be vaccine hesitancy as defined by the SAGE Working Group [19]. Factors related to the hesitancy were investigated with a survey including four categories: (1) sociodemographics, i.e., gender, age, education level, and employment status; (2) health conditions assessed by self-reported health status and any diagnostic chronic illness; (3) experiences related to the virus/vaccines, which covered infection history, providing care to infected persons, knowing someone infected or died from SARS-CoV-2 infection, COVID-19 vaccination doses, side effects after COVID-19 vaccination, and flu vaccination history; and (4) attitudes toward the virus/vaccines.

The fourth category included vaccine confidence and attitudes. Vaccine confidence was measured by four items from the Vaccine Confidence Index. The four items have been tested on Chinese caregivers rating on a five-point Likert scale [26]. Cronbach’s *α* was 0.825 on this four-item scale tested in this study, demonstrating good internal consistency. The total scores were calculated with higher scores indicating higher levels of vaccine confidence. Vaccine attitudes were evaluated by items adapted from the WHO SAGE Vaccine Hesitancy Determinants Matrix Model to assess contextual influences, individual influences, and vaccine-specific issues related to vaccine hesitancy [27]. Contextual influences covered the impact of media/social media and influential leaders and trust in government and pharmaceutical companies. Individual influences included belief in better ways for prevention than vaccines (e.g., developing immunity by getting sick and recovering), perceived knowledge sufficiency, and perceived severity of SARS-CoV-2 infection. Vaccine-specific issues involved vaccine availability in health centers and attitude toward a new vaccine. Each item was treated as an independent variable to assess the association with vaccine hesitancy. This tool was validated by a panel of experts, and the test–retest reliability was satisfactory among dental students (Cohen’s kappa coefficient = 81.83 ± 0.16) [28]. All the sets of measures used in this study were validated by three experts in COVID-19 vaccines to assess their relevance to COVID-19 vaccine hesitancy and the appropriateness for use among the Hong Kong general public.

### 2.4. Data Analysis

The statistical analyses were conducted using the Statistical Package for the Social Sciences (SPSS) version 28 [29] and R 4.4.2 for the age-standardized rate estimation [30]. Descriptive statistics were adopted to characterize the study participants and study responses. Categorical variables were described as frequencies and percentages. Continuous variables are presented as means (standard deviations) for normal distributions or median (*P*_25_, *P*_75_) for skewed distributions. Bivariate analysis was conducted to assess the associations between the primary outcome (i.e., hesitancy of regular COVID-19 vaccination) and independent variables. The chi-squared test was used for categorical independent variables. The *t*-test and Mann–Whitney U test were applied to continuous independent variables for normal and skewed distributions, respectively. Multivariate logistic regression was run with hesitancy as the dependent variable while controlling sociodemographics, health conditions, and other covariates with *p* values < 0.05 in bivariate analysis. Odds ratios (*ORs*) and 95% confidence intervals (95% *CIs*) were estimated. The statistical significance level was set to *p* < 0.05.

## 3. Results

### 3.1. Participant Characteristics

A total of 1213 qualified respondents were interviewed with an effective response rate of 60.2%. The median (*P*_25_, *P*_75_) for the age was 50 (36, 65) years. Among them, 52.9% were female, 65.0% received education at a secondary level or below, and 51.4% were employed. The majority perceived health status as good (60.3%) and reported having none of the chronic illnesses (68.8%). Participant characteristics are presented in Table 1.

### 3.2. Hesitancy of Regular COVID-19 Vaccination

**Figure 1:**
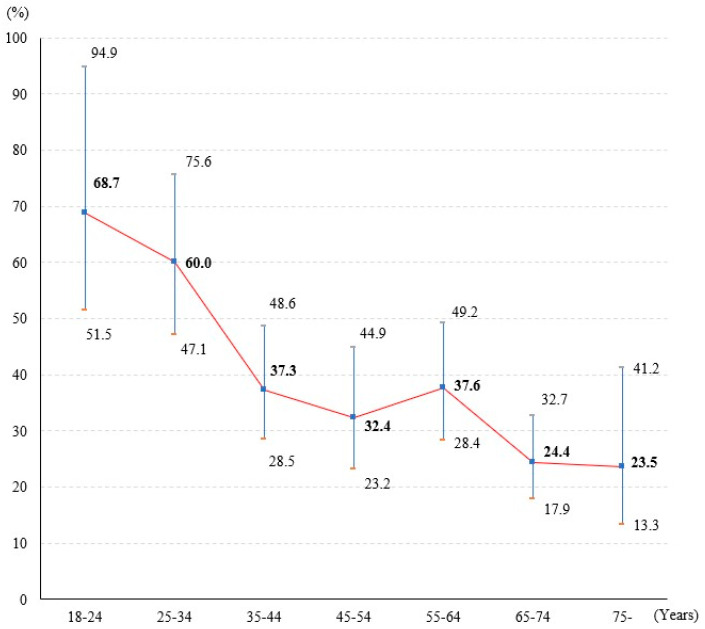
The estimated prevalence of hesitancy for regular COVID-19 vaccination by age groups among Hong Kong adults.

Overall, 43.0% of study participants indicated hesitancy toward regular COVID-19 vaccination annually, of which 26.5% expressed No and 16.5% Not Sure. Age showed a statistically significant association with hesitancy (χ^2^ = 71.264, *p* < 0.01). According to the actual age structure of the Hong Kong population in 2022 [31], the age-standardized hesitancy rate for regular COVID-19 vaccination among Hong Kong adults was estimated to be 39.4% (95% *CI* = 35.3–44.1%). The estimated prevalence of hesitancy among Hong Kong adults presented a sloping S-shape with age groups (Figure 1), with the highest hesitancy among young adults aged 18–24 years (68.7%, 95% *CI* = 51.5–94.9%), followed by decreasing to a lower hesitancy rate among middle-aged adults aged 45–54 years (32.4%, 95% *CI* = 23.2–44.9%), then increasing to a relatively higher point among older adults aged 55–64 years (37.6%, 95% *CI* = 28.4–49.2%), finally decreasing to the lowest hesitancy among elderly adults aged ≥75 years (23.5%, 95% *CI* = 13.3–41.2%).

### 3.3. Determinants of Hesitancy for Regular COVID-19 Vaccination

#### 3.3.1. Bivariate Analysis

Factors of hesitancy for regular COVID-19 vaccination showing statistical significance in univariate analyses are presented in Table 2. Sociodemographics including younger age and higher education level were positively associated with reporting hesitancy. Perceived good health status was associated with lower ratios of hesitancy, while the presence of chronic illness indicated the same relationship. Experiences related to the virus/vaccines, i.e., no SARS-CoV-2 infection history, higher vaccination doses, no vaccination side effects, and flu vaccination history, were linked to less hesitancy. Attitudes toward the virus/vaccines, i.e., higher levels of vaccine confidence, perceived knowledge sufficiency, perceived severity of infection, and trust in pharmaceutical companies and government, were correlated with lower ratios of hesitancy. Beliefs in better ways for prevention than vaccines were linked to higher ratios of hesitancy. Attitudes toward new vaccines and following government advice to take the COVID-19 vaccine were also statistically significantly related to hesitancy; that is, people refusing new vaccines and following government advice out of personal choice reported higher ratios of hesitancy.

#### 3.3.2. Multivariate Analysis

**Figure 2:**
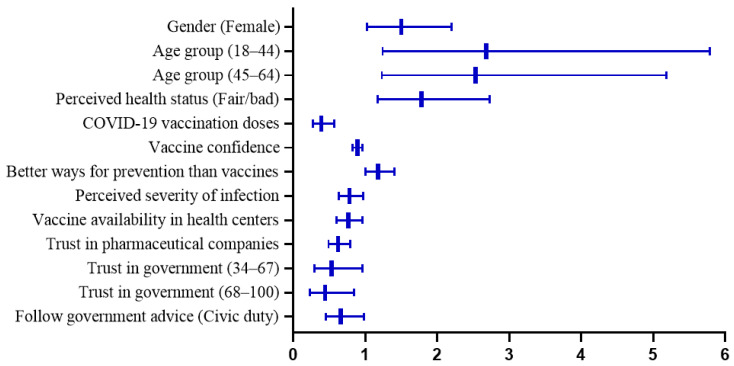
The estimated prevalence of hesitancy for regular COVID-19 vaccination by age groups in Hong Kong adults.

From multivariate logistic regression (Table 3), females were 1.5 times as likely as males to report hesitancy [1.50 (1.02–2.20), *p* = 0.040] after controlling for other factors. Age remained negatively correlated with hesitancy, and higher odds of hesitancy were found among adults aged 18–44 years [2.68 (1.24–5.79), *p* = 0.012] and 45–64 years [2.53 (1.23–5.18), *p* = 0.011] compared to older adults (≥65 years). Participants perceiving fair/bad health status were more likely to be hesitant [1.78 (1.17–2.73), *p* = 0.008]. COVID-19 vaccination doses [0.39 (0.27–0.57), *p* < 0.001], vaccine confidence [0.89 (0.82–0.96), *p* = 0.003], perceived severity of infection [0.78 (0.63–0.97), *p* = 0.026], and vaccine availability in health centers [0.76 (0.60–0.96), *p* = 0.023] remained negative correlates of reporting hesitancy. People believing in better ways for prevention than vaccines showed higher odds of hesitancy [1.18 (1.00–1.40), *p* = 0.048]. Higher levels of trust in pharmaceutical companies [0.62 (0.49–0.79), *p* < 0.001] and trust in government [0.53 (0.29–0.96), *p* = 0.035; 0.44 (0.23–0.84), *p* = 0.013] were negatively associated with lower odds of hesitancy. Following government advice out of civic duty as compared to personal choice was linked to less hesitancy [0.66 (0.45–0.98), *p* = 0.040]. Statistically significant determinants of hesitancy for regular COVID-19 vaccination are presented in Figure 2.

## 4. Discussion

The current random population-based research in Hong Kong is a novel contribution to the body of knowledge on understanding factors associated with vaccine hesitancy within the context of persisting regular COVID-19 vaccination demand. This study is one of the first to estimate the age-standardized COVID-19 vaccine hesitancy rate of Hong Kong adults according to the actual age structure of the local population. Determinants of hesitancy for regular COVID-19 vaccination found in this study included sociodemographics, health status, experiences related to the virus/vaccines, and attitudes toward the virus/vaccines.

From this random population-based telephone survey, we found that 43.0% of the respondents indicated hesitancy toward regular COVID-19 vaccination, corresponding to an estimated hesitancy rate of 39.4% among the whole Hong Kong adult population after we applied age standardization. The hesitancy rate for regular vaccination in our study was slightly higher than the 30.3% reported in a Hong Kong adult population-based online survey conducted during the initial COVID-19 inoculation program, indicating people’s potential declining demand for regular vaccines compared to the basal doses [32]. Despite various government strategies in promoting vaccination in Hong Kong society, hesitancy for regular COVID-19 vaccination has remained high. Indeed, continuous vaccination promotions would be required to reduce future regular COVID-19 vaccine hesitancy. The hesitancy rate for regular vaccines in our study among the general population was also higher when compared to a study conducted on UK healthcare workers (23.5%) [33]. This difference in hesitancy levels toward regular vaccination suggested that the general population is relatively vulnerable to future infection due to lower vaccine acceptance and should be the target of vaccine knowledge transmission. Our findings on the determinants of regular vaccine hesitancy shed light on future programs that can promote regular COVID-19 vaccinations.

The age-standardized regular vaccine hesitancy curve exhibited a sloping S-shape with higher hesitancy among young adults and lower among older adults, which was consistent with previous research on COVID-19 vaccine hesitancy of basal or booster doses [11,14,24]. Our finding echoed the latest statistical data in Hong Kong that young adults (20–29 years) had the lowest booster coverage rate of 79.85% among adults between 20 to 79 years old [9]. Qualitative research revealed that young adults were mostly exposed to fragmented information from social media or self-media, and their main concerns about vaccines were the long-term side effects [34]. Another reason for their higher hesitancy might be attributed to the perception of relatively mild health impacts from COVID-19 on the young [14]. Hence, targeted messaging on vaccine safety and infection risks should reach the young from their main information sources. As for older adults with lower vaccine hesitancy, it might be related to the argument that elderly persons, especially those retired, were more health-conscious [35]. However, older adults aged 80 or above are the least booster vaccinated among adults with a coverage rate of 64.13% [9]. The concerns on side effects of vaccination while experiencing poor health might hinder the oldest old from actual vaccination despite their high willingness to be vaccinated [36]. The oldest old, commonly combined with chronic disease, are vulnerable to severe illness and death from COVID-19 [37]. Hong Kong currently has the longest life expectancy in the world, reaching 85 years, and therefore a significant proportion of the population that may be not fully vaccinated despite the risk of severe complications from the virus [38]. Our observations concur with the current vaccination policy to place the elderly as the top priority for COVID-19 vaccines.

Multivariate regression results demonstrated that gender was an independent determinant of regular COVID-19 vaccine hesitancy with females more likely to be hesitant. Although gender was not identified as a significant predictor of hesitancy in existing studies on regular COVID-19 vaccination [5,16,17], our finding aligned with previous studies on basal or booster doses of vaccines [14,39,40]. Particular concerns from females on COVID-19 vaccines include pregnancy, breastfeeding, and the long-term effects that vaccines may have on offspring [39]. It is important to empower the masses to make informed vaccination decisions underpinned with the knowledge that vaccines are not associated with increased adverse events in pregnancy and will reduce COVID-19-related risks of significant negative outcomes [41,42]. Although earlier studies reported that higher education levels decreased hesitancy toward COVID-19 vaccines, our study echoed recent findings that education level was not a significant predictor for COVID-19 vaccine hesitancy [43,44]. Healthism (personal responsibility for own health and distrust in healthcare institutions) might be one of the drivers of increasing vaccine hesitancy among the high-educated groups, as the emphasis on own judgement and responsibility for health might lead some people to question the necessity of vaccinations [43]. Disciplines of educational degrees might also make contributions (e.g., people who study medical science have more literacy in vaccines and therefore are more likely to accept the vaccinations) to people’s vaccine hesitancy levels [45]. We found that people who perceived themselves as in fair/bad health were more hesitant toward regular vaccines than those who perceived their health as good. The association has not yet been verified in regular vaccine studies and was controversial in some earlier studies for single doses [5,16,17,46,47]. However, a China national scale survey supported our finding [48], which might be attributed to healthcare avoidance traditionally shared by many Chinese people [49]. Surprisingly, the presence of chronic conditions was not identified as a significant predictor of hesitancy for regular COVID-19 vaccination in this study. Our findings highlight the role of subjective perception overriding the objective health condition in determining the act of vaccination. It implied that self-perceived health should be taken into consideration for COVID-19 vaccine promotions. Education is essential to correct misconceptions and make favorable decisions, particularly for those who perceive poor health and have concerns about vaccine-related adverse consequences [48].

For experiences related to the virus/vaccines investigated in this study, only one independent determinant of hesitancy for regular COVID-19 vaccination was significantly identified, i.e., people who took more doses of COVID-19 vaccines tended to be less hesitant. However, influenza vaccination history, which was widely identified as a predictor of COVID-19 vaccine hesitancy for the primary and booster uptake [11,14,50], was not significantly linked to hesitancy for regular COVID-19 vaccination in this study. Our observations suggested that the dosage of COVID vaccine uptake could contribute to understanding people’s readiness for future regular vaccines. Individuals receiving higher doses of COVID-19 vaccines might be more health-conscious and prepared to take regular vaccination as new vaccines are available [14]. In contrast, those with lower vaccine doses will be more reluctant to accept regular vaccines, which favors a higher risk of infection and severe outcomes for lack of vaccine defense [51,52]. Much attention should be attached to the low vaccination group with targeted studies and evidence-based interventions. Previous research indicated that having COVID-19 vaccination side effects was associated with the vaccine hesitancy of basal or booster doses [11,14,40]. However, experiencing vaccination side effects was not a significant independent determinant of hesitancy for regular COVID-19 vaccination in our study and therefore should be examined in future studies.

Most attitudinal elements, including vaccine confidence, beliefs in better ways for prevention, perceived severity and availability, and trust in pharmaceutical companies and government, were identified as independent determinants of hesitancy for regular COVID-19 vaccination. In particular, people who have higher levels of vaccine confidence were significantly less likely to be hesitant to take regular COVID-19 vaccines. Vaccine confidence has been consistently validated as a key determinant of COVID-19 vaccine hesitance for basal, booster, or regular doses in many studies [16,23,53,54], as well as in our study. The components of vaccine confidence, including vaccine importance, safety, efficacy, and value compatibleness, deserve to be highlighted in vaccine promotion campaigns to improve the future uptake of regular vaccines. In addition, we found that people who believed there were better ways for preventing COVID-19 than vaccines, e.g., developing immunity by getting sick and recovering, were significantly more likely to be hesitant to take regular COVID-19 vaccines. This association was consistent with previous studies of COVID-19 vaccine hesitancy for single does and was first verified with a pattern of regular uptake in our study [27,28]. Indeed, clinical research indicated that adverse consequences related to SARS-CoV-2 infection outweighed the side effects of vaccination [55,56]. This further reinforces the need for educational programs on vaccine literacy in order to correct people’s misconceptions about the pros and cons in relation to getting infected vs. taking the vaccines [57].

The perceived severity of SARS-CoV-2 infection was significantly associated with decreased hesitancy toward regular vaccination, which has not been explored in previous studies. This again calls for education on the advantages of vaccination outweighing the consequences of getting infected. Notably, the infection history of SARS-CoV-2 was not found to be significantly related to hesitancy for regular COVID-19 vaccination. This might be because people are not aware of the negative consequences of repetitive infections, and they might also not be aware that virus mutations might weaken the effects of natural immunity gained from previous infection histories [58,59]. Thus, educational programs also need to address this important point of the necessity of taking vaccines even in those with infection histories. In addition, the negative consequences of SARS-CoV-2 infection, which might cause serious health problems and possible long-term health problems, need to be highlighted in vaccination promotions [51,52,60]. Perceived vaccine availability in health centers was also identified as a significant determinant linked to reduced regular vaccine hesitancy in our study. The sufficient resources for vaccine production or purchases need to be addressed by the government, and relevant policies need to be developed to cope with the potential threats of future pandemics over the long term.

Importantly, the level of trust in relevant key stakeholders, i.e., pharmaceutical companies and government, was a significant promoting factor of decreased hesitancy for receiving regular COVID vaccination. Owing to the immediate and immense demand for the COVID-19 vaccine, an array of pharmaceutical companies entered the market [61]. However, the accelerated vaccine rollout, lack of knowledge about the development process, and the influx of too many manufacturers were likely to undermine the trust in pharmaceutical companies, which in turn raised vaccine hesitancy as a result [24]. Therefore, transparent information about the development process of vaccines offered by government-permitted suppliers should be exposed to the general public. Government plays a leading role in vaccine-related policies and promotions. We found that people with higher levels of trust in the government were less hesitant toward the uptake of regular vaccines. Also, our study indicated that individuals who perceived it was a civic duty to follow the government’s advice to take vaccines had significantly lower hesitancy of regular vaccination than those who perceived it as a personal choice. This finding was aligned with a survey of UK adults, which revealed that protecting the health of others was a key facilitator for considering regular booster uptake [16]. In particular, the social norm that encourages Chinese people to prioritize collective benefits and make contributions to society would contribute to a sense of civic duty and motivate them to take vaccines [62]. For future vaccination promotion programs, it would be necessary to emphasize that being vaccinated not only benefits individuals but also protects the health of others such as family, friends, and vulnerable groups. The civic responsibilities of each citizen in promoting the health of all members of society should be highlighted.

This study is the first representative population-based survey in Hong Kong that evaluated the associations between sociodemographics, health status, experiences related to the virus/vaccines, attitudes toward the virus/vaccines, and the hesitancy of regular COVID-19 vaccination. The findings were built on the foundation of a random sampling survey with a highly effective response rate. There are, nevertheless, several limitations that should be considered when interpreting the study findings. Firstly, the cross-sectional design adopted in this study restricted the ability to draw causal inferences from the examined factors. Also, the findings were only cross-sectional rather than longitudinal and cannot reveal the changing trend of people’s attitudes toward regular COVID-19 vaccination. Secondly, this is a population-based survey of Hong Kong adults; thus, the generalizability of our findings to other settings and age groups is dubious. Thirdly, there is a possibility of non-response bias, despite attempts that were made to minimize the non-response rate. Finally, we adopted a quantitative survey design without qualitative interviews, which might have hindered the exploration of people’s underlying thoughts and notions on regular COVID-19 vaccination. Nonetheless, our study is a random population-based survey with a large sample and evaluated the prevalence and determinants of hesitancy for regular COVID-19 vaccination as guided by relevant theory and a comprehensive literature review.

Our findings may have implications not only for the vaccine policymakers in government health sectors but also for vaccine program promoters with community healthcare professionals who are often the most accessible and trusted sources of health guidance. First of all, tailored education is essential to address the high level of hesitancy of the general population toward future regular COVID-19 vaccination. In particular, the target message on COVID-19 risks and vaccine safety should reach the population most at risk of hesitancy, including females, young adults, and people perceiving poor health and receiving fewer doses of COVID-19 vaccines, so as to correct misinformation and make informed decisions. In addition, updated education (e.g., efficacy and safety of new COVID-19 vaccines, consequences of repetitive infections, and long-term effects of infections) should be adopted to increase vaccine literacy and confidence as well as improve trust in manufacturers and the government. Civic duty could also be appealed to as a means of encouraging the public to follow the government’s suggestions for vaccine uptake. Moreover, longitudinal design and the inclusion of qualitative interviews are recommended for future studies to examine causal effects and explore people’s subjective perceptions in depth.

## 5. Conclusions

This random population-based study provides preliminary evidence on the regular COVID-19 vaccination readiness and determinants. There is still a large proportion of people who are hesitant toward future regular COVID-19 vaccination in Hong Kong. The population subgroups of females, young adults, self-perceived fair/bad health, and people receiving fewer doses of COVID-19 vaccines should be considered at most risk of hesitancy and therefore may benefit from targeted educational campaigns. Our findings also highlight the influence of attitudes on regular vaccine uptake. This knowledge will contribute to the design of effective interventions that should incorporate attitude-change strategies to improve vaccine confidence, enhance perceptions of severity, improve vaccine availability, and build trust in both manufacturers and the government. Our findings provide directions not only for regular vaccination coping with potential waves of COVID-19 outbreaks but also for new vaccines that will be developed for future pandemics.

## Figures and Tables

**Table 1:** Participant characteristics (*n* = 1213).

	*n*	%		*n*	%
Gender			Education level		
Male	571	47.1	Primary or below	226	18.8
Female	642	52.9	Secondary	558	46.2
			College or above	422	35.0
Age group			Employment		
18–24	85	8.1	Unemployed	587	48.6
25–34	144	13.7	Employed	620	51.4
35–44	193	18.5	Health condition		
45–54	164	15.7	Good	730	60.3
55–64	176	16.9	Fair	407	33.6
65–74	190	18.2	bad	73	6.1
≥75	93	8.9	Chronic illness		
Age			No	834	68.8
*M* (*P*_25_, *P*_75_)	50 (36, 65)		Yes	379	31.2

Note. The sample size varied due to missing data.

**Table 2:** Factors associated with hesitancy of regular COVID-19 vaccination in bivariate analysis.

Factors		Regular Vaccination		*χ*^2^/*Z*	*p*
		No/Not Sure	Yes		
Sociodemographics					
Gender	Male	240 (42.2)	329 (57.8)	0.311	0.577
	Female	281 (43.8)	361 (56.2)		
Age	18–44	218 (51.7)	204 (48.3)	41.661	<0.001
	45–64	127 (37.5)	212 (62.5)		
	≥65	79 (27.9)	204 (72.1)		
Education level	Primary or below	71 (31.4)	155 (68.6)	28.537	<0.001
	Secondary	227 (40.8)	329 (59.2)		
	College or above	221 (52.4)	201 (47.6)		
Employment	Unemployed	237 (40.4)	349 (59.6)	2.820	0.093
	Employed	280 (45.2)	339 (54.8)		
Health conditions					
Perceived health status	Good	208 (38.5)	448 (61.5)	14.900	<0.001
	Fair/bad	239 (49.7)	242 (50.3)		
Chronic illness	No	383 (46.0)	450 (54.0)	9.728	0.002
	Yes	138 (36.4)	241 (63.6)		
Experiences					
SARS-CoV-2 infection	No	342 (41.1)	491 (58.9)	8.179	0.017
	Yes	152 (45.4)	183 (54.6)		
	Probably	26 (61.9)	16 (38.1)		
COVID-19 vaccination doses	Zero	64 (86.5)	10 (13.5)	173.685	<0.001
	One	42 (67.7)	20 (32.3)		
	Two	264 (54.8)	218 (45.2)		
	Three	151 (25.5)	441 (74.5)		
COVID-19 vaccination side effects	None	197 (33.7)	388 (66.3)	25.939	<0.001
	Mild to moderate	228 (45.2)	276 (54.8)		
	Moderate to severe	26 (65.0)	14 (35.0)		
Flu vaccination	No	301 (48.9)	314 (51.1)	60.632	<0.001
	Yes	212 (36.4)	370 (63.6)		
Attitudes					
Vaccine confidence		13 (10, 16)	16 (14, 18)	−15.177	<0.001
Better ways for prevention than vaccines		4 (3, 4)	3 (2, 4)	9.665	<0.001
Perceived knowledge sufficiency		3 (3, 4)	4 (3, 4)	−10.915	<0.001
Perceived severity of infection		2 (2, 3)	3 (2, 3)	−2.098	0.036
Vaccine availability in health centers		4 (3, 4)	4 (4, 5)	−8.618	<0.001
Trust in pharmaceutical companies		3 (2, 4)	4 (3, 4)	−11.824	<0.001
Trust in government	0–33	181 (76.7)	55 (23.3)	204.240	<0.001
	34–67	223 (46.9)	252 (53.1)		
	68–100	103 (21.4)	378 (78.6)		
Follow government advice	Personal choice	339 (69.9)	146 (30.1)	63.675	<0.001
	Civic duty	302 (46.2)	352 (53.8)		
Attitude toward new vaccine	First to get	14 (2.7)	105 (82.3)	86.725	<0.001
	Wait and see	441 (44.0)	561 (56.0)		
	Refuse	59 (78.7)	16 (21.3)		

Note. The sample size varied due to missing data.

**Table 3:** Factors associated with hesitancy of regular COVID-19 vaccination in multivariate logistic regression.

Factors		*OR*	95% *CI*	*p*
Sociodemographics				
Gender	Male	Reference		
	Female	1.50	1.02–2.20	0.040
Age	≥65	Reference		
	45–64	2.53	1.23–5.18	0.011
	18–44	2.68	1.24–5.79	0.012
Education level	Primary or below	Reference		
	Secondary	1.41	0.70–2.82	0.332
	College or above	1.56	0.72–3.37	0.258
Employment	Unemployed	Reference		
	Employed	0.84	0.55–1.29	0.424
Health conditions				
Perceived health status	Good	Reference		
	Fair/bad	1.78	1.17–2.73	0.008
Chronic illness	No	Reference		
	Yes	1.21	0.73–2.01	0.468
Experiences				
COVID-19 vaccination doses		0.39	0.27–0.57	<0.001
COVID-19 vaccination side effects	None	Reference		
	Mild to moderate	1.07	0.72–1.59	0.748
	Moderate to severe	1.63	0.40–6.59	0.492
SARS-CoV-2 infection	No	Reference		
	Yes	0.69	0.43–1.10	0.118
Flu vaccination	No	Reference		
	Yes	0.76	0.52–1.12	0.171
Attitudes				
Vaccine confidence		0.89	0.82–0.96	0.003
Better ways for prevention than vaccines		1.18	1.00–1.40	0.048
Perceived knowledge sufficiency		1.05	0.82–1.34	0.704
Perceived severity of infection		0.78	0.63–0.97	0.026
Vaccine availability in health centers		0.76	0.60–0.96	0.023
Trust in pharmaceutical companies		0.62	0.49–0.79	<0.001
Trust in government	0–33	Reference		
	34–67	0.53	0.29–0.96	0.035
	68–100	0.44	0.23–0.84	0.013
Follow government advice	Personal choice	Reference		
	Civic duty	0.66	0.45–0.98	0.040
Attitude toward a new vaccine	First to get	Reference		
	Wait and see	1.28	0.59–2.78	0.536
	Refuse	1.86	0.46–7.53	0.385

## Data Availability

The data that support the findings of this study are available from the corresponding author upon reasonable request.
